# Temporal Kinetics of RNAemia and Associated Systemic Cytokines in Hospitalized COVID-19 Patients

**DOI:** 10.1128/mSphere.00311-21

**Published:** 2021-05-28

**Authors:** Debby van Riel, Carmen W. E. Embregts, Gregorius J. Sips, Johannes P. C. van den Akker, Henrik Endeman, Els van Nood, Mathijs Raadsen, Lisa Bauer, Jeroen van Kampen, Richard Molenkamp, Marion Koopmans, David van de Vijver, Corine H. GeurtsvanKessel

**Affiliations:** a Department of Viroscience, Erasmus MC, Rotterdam, The Netherlands; b Medical Microbiology and Infectious Diseases, Erasmus MC, Rotterdam, The Netherlands; c Department of Intensive Care, Erasmus MC, Rotterdam, The Netherlands; University of Maryland School of Medicine

**Keywords:** COVID-19, SARS-CoV-2, RNAemia, inflammatory cytokines, extrarespiratory, IL-6, MCP-1, IL-10, viral load, pathogenesis, cytokine storm, interferon

## Abstract

COVID-19 is associated with a wide range of extrarespiratory complications, of which the pathogenesis is currently not fully understood. However, both systemic spread and systemic inflammatory responses are thought to contribute to the systemic pathogenesis. In this study, we determined the temporal kinetics of viral RNA in serum (RNAemia) and the associated inflammatory cytokines and chemokines during the course of COVID-19 in hospitalized patients. We show that RNAemia can be detected in 90% of the patients who develop critical disease, compared to 50% of the patients who develop moderate or severe disease. Furthermore, RNAemia lasts longer in patients who develop critical disease. Elevated levels of interleukin-10 (IL-10) and MCP-1—but not IL-6—are associated with viral load in serum, whereas higher levels of IL-6 in serum were associated with the development of critical disease. In conclusion, RNAemia is common in hospitalized patients, with the highest frequency and duration in patients who develop critical disease. The fact that several cytokines or chemokines are directly associated with the presence of viral RNA in the circulation suggests that the development of RNAemia is an important factor in the systemic pathogenesis of COVID-19.

**IMPORTANCE** Severe COVID-19 can be considered a systemic disease as many extrarespiratory complications occur. However, the systemic pathogenesis is poorly understood. Here, we show that the presence of viral RNA in the blood (RNAemia) occurs more frequently in patients who develop critical disease, compared to patients with moderate or severe disease. In addition, RNAemia is associated with increased levels of inflammatory cytokines and chemokines, like MCP-1 and IL-10, in serum during the course of disease. This suggests that extrarespiratory spread of SARS-CoV-2 contributes to systemic inflammatory responses, which are an important factor in the systemic pathogenesis of COVID-19.

## OBSERVATION

COVID-19 has been associated with a wide range of extrarespiratory complications, including neurologic, cardiac, and thromboembolic complications. The pathogenesis of these extrarespiratory complications is not fully understood, but several mechanisms are thought to contribute, including the systemic spread of SARS-CoV-2 and systemic inflammatory cytokines (1, 2). Even though SARS-CoV-2 viral RNA and inflammatory cytokines have been detected in the blood, their kinetics during infection are poorly understood, and it is unclear if either RNAemia or inflammatory cytokines are associated with each other or with other disease parameters. We hypothesize that RNAemia occurs frequently during severe COVID-19 and that it contributes to the systemic inflammatory responses. Therefore, we aimed to get insight into the kinetics of SARS CoV-2 RNAemia and associated systemic inflammatory response during the course of COVID-19.

Diagnostic specimens of 20 patients (16 male, 4 female), hospitalized at the Erasmus MC in The Netherlands in March and April 2020, were analyzed. Ten patients developed moderate or severe disease (grouped together), and 10 developed critical disease, according to NIH disease severity guidelines (https://www.covid19treatmentguidelines.nih.gov/overview/management-of-covid-19). One or more underlying diseases were present in 19 patients, which included cardiovascular diseases (11), diabetes (9), previous cerebrovascular accident (4), previous respiratory disease (3), cancer (3), solid organ transplantation (2), kidney failure (1), psychosis (1), or sarcoidosis (1). None of the patients received dexamethasone during the course of disease. Further patient characteristics are included in Table S1 in the supplemental material and described in Text S1 (supplemental materials and methods). A total of 176 serum samples were analyzed, and a paired respiratory tract (RT) sample ([naso]pharyngeal swab or sputum) was available for 131 specimens (Table S2). For cytokine and chemokine analyses, sera of 18 healthy control donors were included (Table S1).

10.1128/mSphere.00311-21.1 TEXT S1 Supplemental materials and methods. Download Text S1, DOCX file, 0.02 MB. Copyright © 2021 van Riel et al. 2021 van Riel et al. https://creativecommons.org/licenses/by/4.0/ This content is distributed under the terms of the Creative Commons Attribution 4.0 International license.

10.1128/mSphere.00311-21.5 TABLE S1 Information on patients and healthy donors included in the study. Download Table S1, DOCX file, 0.01 MB. Copyright © 2021 van Riel et al. 2021 van Riel et al. https://creativecommons.org/licenses/by/4.0/ This content is distributed under the terms of the Creative Commons Attribution 4.0 International license.

10.1128/mSphere.00311-21.6 TABLE S2 Detection of viral RNA in serum of patients with moderate/severe or critical disease. Analyses are done on the total number of samples and on samples from 1 to 10 days post-disease onset (dpd) and >10 dpd. Download Table S2, DOCX file, 0.01 MB. Copyright © 2021 van Riel et al. 2021 van Riel et al. https://creativecommons.org/licenses/by/4.0/ This content is distributed under the terms of the Creative Commons Attribution 4.0 International license.

**FIG 1:**
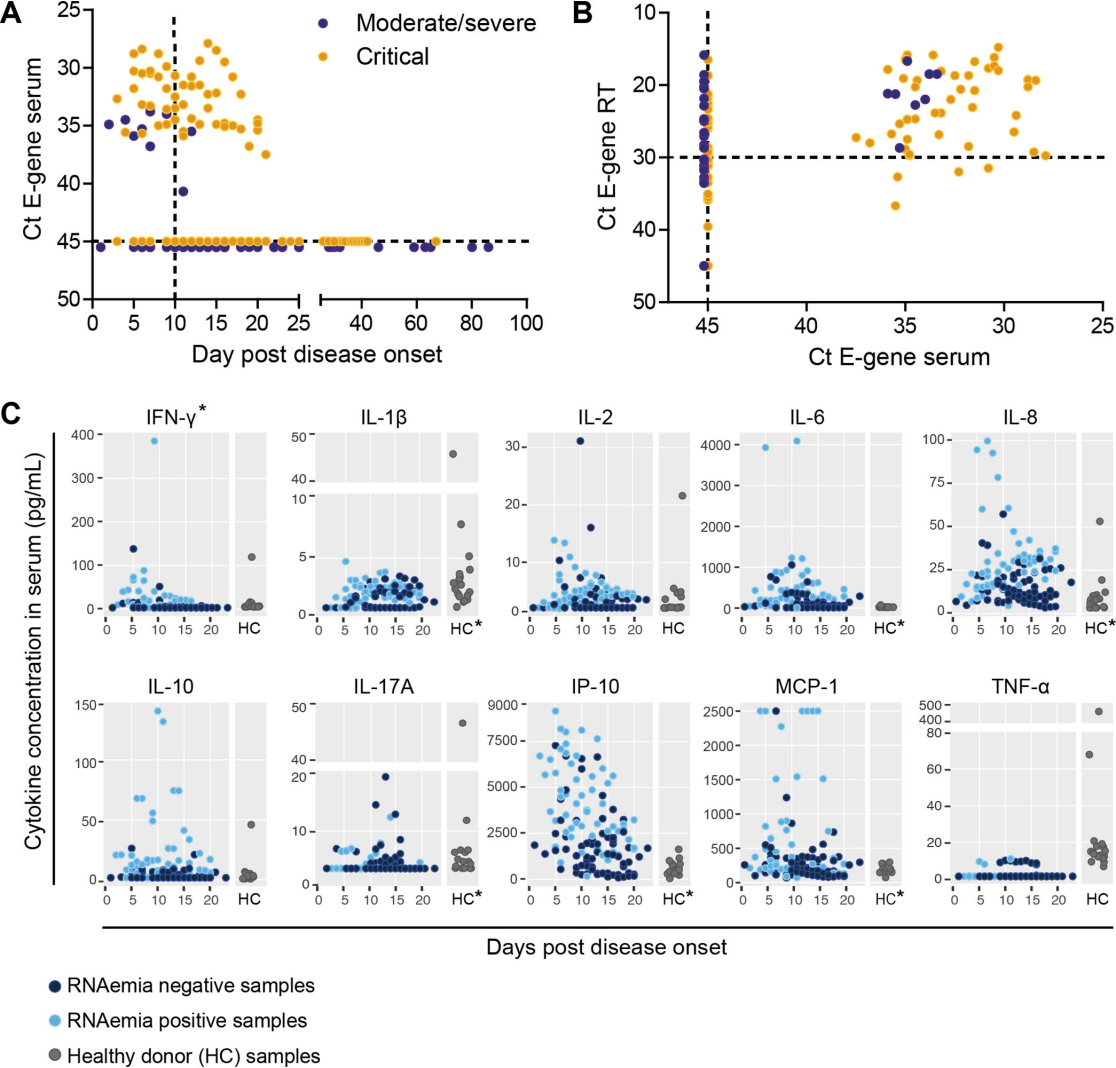
RNAemia and systemic inflammatory responses in hospitalized COVID-19 patients. (A) The detection of SARS-CoV-2 viral RNA (E gene) in serum in patients with moderate/severe or critical disease plotted against the day post-disease onset. (B) The detection of SARS-CoV-2 viral RNA in serum plotted against the detection of SARS-CoV-2 viral RNA in respiratory sample. The dashed line represents a *C_T_* value of 30 in respiratory samples. (C) Individual cytokines plotted against the day post-disease onset. Serum cytokine levels of healthy control donors (HC, *n* = 18) are shown on the right of each individual cytokine graph. Asterisks (*) at the top of the graphs refer to significantly higher levels in multivariate analyses <10 dpd compared to >10 dpd, whereas asterisks (*) below the graphs indicate significant difference in cytokine levels between healthy control donors and COVID-19 patients.

Viral RNA was measured by quantitative PCR (qPCR) for the E gene in all serum and RT samples as described previously (3). RNAemia was detected in 50% and 90% of the patients with moderate/severe or critical disease, respectively. The duration of the RNAemia per patient could not be determined, since samples early after disease onset were not available for all patients. RNAemia was more frequently detected from 11 days post-disease onset (dpd) in patients who developed critical disease (odds ratio [OR] 4.65; confidence interval [CI], 1.05 to 20.64; *P* = 0.038; Fig. 1A and Table S2), as calculated using generalized estimated equations, corrected for repeated samples within patients. Furthermore, independent of disease severity, RNAemia was associated with a threshold cycle (*C_T_*) value of <30 in paired RT samples (OR, 9.47; CI, 3.08 to 29.07; *P* = 0.009) and <11 dpd (OR, 4.61; CI, 1.80 to 11.87; *P* = 0.001; Fig. 1B). In this study, there was no correlation between RNAemia and age (OR, 1.0; CI, 0.99 to 1.01; *P* = 0.31) or body mass index (BMI) (OR, 1.0; CI, 0.9 to 1.2; *P* = 0.58). Virus could not be isolated from serum samples, but since these had been collected for molecular and serological diagnostics, the sample handling and storage were most likely suboptimal for virus culture. In addition, we have previously shown that virus is difficult to culture from samples with a *C_T_* value above 27 (4).

Next, we used a 13-plex cytometric bead assay to determine the kinetics of interleukin-1β (IL-1β), IL-2, IL-4, IL-6, IL-8, IL-10, IL-12p70, IL-17A, IP-10, gamma interferon (IFN-γ), tumor necrosis factor alpha (TNF-α), MCP-1, and transforming growth factor β (TGF-β) in serum of patients up to 21 dpd (Text S1 and Fig. S1). All tested cytokines were significantly regulated during the course of infection, with the exception of IL-4, IL-12p70, and TGF-β, which were therefore excluded from further analysis. IFN-γ, IL-1β, IL-2, IL-6, IL-8, IL-10, IL-17A, IP-10, MCP-1, and TNF-α were induced during the course of disease (Fig. 1C), although large differences were observed among individual patients. Overall, a dynamic response was observed in the majority of patients (Fig. S1). In COVID-19 patients, IL-1β, IL-6, IL-8, IL-17A, IP-10, and MCP-1 levels differed significantly from the healthy control group (Fig. 1C and Table S3). Generalized estimated equations, corrected for repeated sampling within patients, were used to determine associations of individual cytokines with RNAemia, an RT *C_T_* of <30, disease severity and outcome, and period after disease onset. Of all cytokines and chemokines, only IFN-γ was associated with a disease period of ≤10 days in both univariate and multivariate analysis (Table 1 and Fig. 1C).

**TABLE 1:** Concentrations of individual cytokines and associations with RNAemia, a *C_T_* value in respiratory samples of <30, development of critical disease, fatal disease outcome, and the first 10 days after disease onset^*a*^

Cytokine	Median concn, pg/ml (range)		Univariate *P* value	Multivariate *P* value^*b*^
	Association with RNAemia			
	*C_T_* < 45 in serum (13 [58])	*C_T_* = 45 in serum (19 [72])		
IFN-γ	3.0 (3–385)	3.0 (3–138)	0.003	0.528
IL-1β	2.0 (0.8–4.8)	0.8 (0.8–3.5)	0.046	0.099
IL-2	4.2 (2.1–13.9)	2.1 (2.1–31.1)	0.059	0.969
IL-6	203 (10–4,090)	61 (8–1,055)	0.018	0.103
IL-8	25.4 (4.6–99.8)	11.7 (3.4–57.5)	<0.001	0.251
IL-10	11.5 (1.7–140)	1.7 (1.7–26.2)	<0.001	**<0.001**
IL-17A	3.2 (3.2–13.3)	3.2 (3.2–19.4)	0.150	
IP-10	3,683 (168–9,155)	1,380 (86–7,282)	<0.001	0.170
MCP-1	272 (73–2,500)	184 (70–2,500)	0.019	**0.036**
TNF-α	1.2 (1.2–10.7)	1.2 (1.2–9.8)	0.410	
	Association with a high viral load (*C_T_* < 30) in respiratory tract			
	*C_T_* < 30 in RT (18 [82])	*C_T_* ≥ 30 in RT (15 [32])		
IFN-γ	3.0 (3–385)	3.0 (3–11.5)	0.007	**0.015**
IL-1β	1.8 (0.8–4.8)	0.8 (0.8–2.8)	0.008	**<0.001**
IL-2	3.8 (2.1–13.9)	2.1 (2.1–16.1)	0.014	0.686
IL-6	143 (10–4,090)	82 (8–768)	0.029	0.749
IL-8	20 (4.4–99.8)	11.7 (3.4–40.8)	0.002	0.269
IL-10	6.4 (1.7–141)	1.7 (1.7–9.7)	0.003	0.271
IL-17A	3.2 (3.2–19.4)	3.2 (3.2–13.8)	0.670	
IP-10	2,686 (86–9,155)	1,123 (123–6,656)	0.021	0.759
MCP-1	239 (79–2,500)	154 (73–2,500)	<0.001	0.358
TNF-α	1.2 (1.2–9.8)	1.2 (1.12–10.7)	0.823	
	Association with the development of critical disease			
	Critical disease (10 [91])	Moderate or severe disease (10 [39])		
IFN-γ	3.0 (3–73)	6.0 (3–385)	0.084	0.228
IL-1β	2.1 (0.8–4.8)	0.8 (0.8–2.0)	0.003	**0.003**
IL-2	3.6 (2.1–13.9)	2.2 (2.1–31.1)	0.440	
IL-6	144 (10–4,090)	49.8 (8.1–254)	0.041	**0.005**
IL-8	19.4 (3.4–99.8)	12.4 (14.5–29.9)	0.140	
IL-10	5.7 (1.7–141)	5.7 (1.7–67.9)	0.620	
IL-17A	3.2 (3.2–19.4)	3.2 (3.2–14.9)	0.700	
IP-10	1,918 (86–9,155)	2,487 (692–8,015)	0.121	
MCP-1	197 (73–2,500)	316 (70–2,500)	0.480	
TNF-α	1.2 (1.2–10.7)	1.2 (1.2–9.7)	0.373	
	Association with fatal outcome			
	Survivor (15 [80])	Nonsurvivor (5 [50])		
IFN-γ	3.0 (3–385)	3 (3–73)	0.880	
IL-1β	0.8 (0.8–3.5)	1.9 (0.8–4.8)	0.157	
IL-2	2.3 (2.1–31.1)	4.0 (2.1–13.9)	0.149	
IL-6	56 (8–4,090)	270 (10–3,930)	0.031	0.063
IL-8	13.4 (3.4–60.9)	23.9 (4.6–99.8)	0.070	0.607
IL-10	3.3 (1.7–140.8)	6.6 (1.7–67.9)	0.610	
IL-17A	3.2 (3.2–14.9)	3.2 (3.2–19.4)	0.210	
IP-10	1,922 (86–8,015)	2,601 (168–9,155)	0.160	
MCP-1	217 (70–2,500)	247 (73–2,500)	0.470	
TNF-α	1.2 (1.2–9.7)	1.2 (1.2–10.7)	0.930	
	Association with the first 10 days post-disease onset			
	≤10 days (14 [51])	>10 days (18 [79])		
IFN-γ	6 (3–385)	3 (3–31.1)	<0.001	**0.034**
IL-1β	1.3 (0.8–4.8)	1.8 (0.8–3.88)	0.330	
IL-2	3.1 (2.1–31.1)	3.5 (2.1–16.1)	0.690	
IL-6	144 (10–3,930)	74 (8–4,090)	0.147	
IL-8	17 (4.8–99.8)	14.7 (3.4–60.9)	0.360	
IL-10	7 (1.7–140.8)	1.8 (1.7–131.9)	0.182	
IL-17A	3.2 (3.2–6.1)	3.2 (3.2–19.4)	0.400	
IP-10	3,683 (252–9,155)	1,653 (86–7,646)	0.013	0.058
MCP-1	320 (70–2,500)	170 (73–2,500)	0.190	
TNF-α	1.2 (1.2–9.8)	1.2 (1.2–10.7)	0.800	

a Values with the group names indicate the number of patients and the number of samples (patients [number of samples]) within the specified group. Univariate generalized estimated equations were performed on the individual cytokines (log_10_ transformed), and associations with a *P* value of <0.1 were included in a multivariate analysis.

b Values in bold are statistically different (*P* < 0.05) in the multivariate analyses.

10.1128/mSphere.00311-21.2 FIG S1 Individual patient data of days post-disease onset, *C_T_* value in the respiratory tract (RT), *C_T_* value in serum, and the concentration of respective cytokines in pg/ml. Download FIG S1, TIF file, 0.5 MB. Copyright © 2021 van Riel et al. 2021 van Riel et al. https://creativecommons.org/licenses/by/4.0/ This content is distributed under the terms of the Creative Commons Attribution 4.0 International license.

10.1128/mSphere.00311-21.7 TABLE S3 Concentrations of individual cytokines in serum of healthy donors and univariate analysis of cytokine concentrations in serum of healthy donors compared to SARS-CoV-2 patients, moderate/severe patients, critical patients, and RNAemia-positive serum samples. Univariate generalized estimated equations were performed on the individual cytokines (log_10_ transformed) and differences with a *P* value of <0.05 were regarded as significant. Table S3, DOCX file, 0.02 MB Copyright © 2021 van Riel et al. 2021 van Riel et al. https://creativecommons.org/licenses/by/4.0/ This content is distributed under the terms of the Creative Commons Attribution 4.0 International license.

RNAemia was associated with increased levels of IL-1β, IL-6, IL-8, IL-10, IP-10, and MCP-1 in a univariate analysis. Subsequent multivariate analyses showed that IL-10 and MCP-1 were independently associated with RNAemia (Table 1 and Fig. S2A). In a univariate analysis, a *C_T_* value of <30 in the RT was associated with increased serum levels of IFN-γ, IL-1β, IL-2, IL-6, IL-8, IL-10, IP-10, and MCP-1 compared to a *C_T_* value of ≥30 in the RT and with increased levels of IFN-γ and IL-1β in a multivariate analysis (Table 1 and Fig. S2B).

10.1128/mSphere.00311-21.3 FIG S2 Scatterplots of all individual cytokine or chemokine measurements, plotted against the presence of RNAemia in serum (A) or a *C_T_* in the respiratory tract of <30 (B). Samples are divided between 1 to 10 days post disease onset (dpd) (light blue) and >10 dpd (dark blue). Asterisks (*) indicate significant differences (*P* < 0.05) in a multivariate analysis. Download FIG S2, TIF file, 0.7 MB. Copyright © 2021 van Riel et al. 2021 van Riel et al. https://creativecommons.org/licenses/by/4.0/ This content is distributed under the terms of the Creative Commons Attribution 4.0 International license.

Patients who developed critical disease had significantly higher levels of IL-1β and IL-6 than patients who developed moderate or severe disease (Table 1 and Fig. S3A). While leukocyte counts in critical patients were significantly higher than in patients who developed moderate or severe disease (Table S2), observed differences were minor and both patient groups showed median leukocyte counts that fall within the normal range. Death as an outcome was not associated with elevated levels of any of the cytokines included in this study in a multivariate analysis (Table 1 and Fig. S3B).

10.1128/mSphere.00311-21.4 FIG S3 Scatterplots of all individual cytokine or chemokine measurements, plotted against the severity of disease (A) or the outcome of disease (B). Samples are divided between 1 to 10 dpd (light blue) and >10 dpd (dark blue). Asterisks (*) indicate significant difference (*P* < 0.05) in a multivariate analysis. Download FIG S3, TIF file, 0.7 MB. Copyright © 2021 van Riel et al. 2021 van Riel et al. https://creativecommons.org/licenses/by/4.0/ This content is distributed under the terms of the Creative Commons Attribution 4.0 International license.

Altogether, this study shows a high prevalence of RNAemia in hospitalized COVID-19 patients. The overall detection of RNAemia in 70% of patients included in this study is higher than in most other reports (5–8). This is likely due to the fact that all patients included in this study were hospitalized with moderate, severe, or critical disease and that we analyzed samples throughout the course of disease. The fact that RNAemia is detected at later time points post-disease onset in patients who develop critical disease, together with the detection of SARS-CoV-2 in extrarespiratory tissues such as the heart, liver, and kidney (1, 9, 10), suggests that systemic spread of SARS-CoV-2 contributes to the pathogenesis of severe COVID-19. The origin of the viral RNA detected in the blood is not known, but high titers in the respiratory tract, in combination with the histological evidence for severe damage in the lungs (11, 12), suggest direct spillover of virus from the lung into the circulation.

The role of systemic cytokines during the course of COVID-19 is only partly understood. Elevated cytokine responses are to be expected during viral infections, but if and how protective responses transform to uncontrolled inflammation that contributes to the development of COVID-19 are unclear (13). For example, others and our study show that elevated levels of IL-6 are associated with the development of more severe disease (8, 14–16), whereas absolute concentrations of IL-6 are relatively low in COVID-19 patients compared to patients with acute respiratory distress syndrome (ARDS), sepsis, or cytokine release syndrome (17).

Systemic cytokine profiles are diverse among patients and vary during the course of disease, and exact triggers are unknown. We show that IL-10 and MCP-1 are associated with the presence of RNAemia, while IL-1β and IL-6 are associated with the development of critical disease. This suggests that virus spread beyond the respiratory tract contributes to—at least part of—the systemic cytokine responses. Up to the present, the beneficial effect of immunotherapies to modulate the systemic immune response, including tocilizumab (monoclonal antibody against IL-6) and anakinra (recombinant interleukin-1 receptor antagonist), do not consistently find a positive effect on morbidity and mortality (18–20). Most likely, the effect of these immunotherapies depends on many factors including the diversity among patients and timing. It is therefore important to acquire more insight into the pathogenesis of these systemic responses, the diversity among patients, and the temporal kinetics of systemic responses in order to develop personalized intervention strategies.

Even though this study has several limitations, such as the number of patients, the retrospective character, and the usage of diagnostic samples, it reveals important insight into the temporal kinetics of both RNAemia and associated systemic cytokine responses. Our findings suggest that both RNAemia and systemic responses contribute—at least in part—to the systemic pathogenesis of severe COVID-19, which fits with the wide spectrum of extrarespiratory complications associated with COVID-19. Furthermore, we show that in order to acquire more insights into the systemic pathogenesis, it is essential to analyze the kinetics of systemic viral load and responses during the course of disease. This knowledge will be essential for the development and timing of intervention strategies that target either the host immune response or virus replication.

## References

[1] Gupta A, Madhavan MV, Sehgal K, Nair N, Mahajan S, Sehrawat TS, Bikdeli B, Ahluwalia N, Ausiello JC, Wan EY, Freedberg DE, Kirtane AJ, Parikh SA, Maurer MS, Nordvig AS, Accili D, Bathon JM, Mohan S, Bauer KA, Leon MB, Krumholz HM, Uriel N, Mehra MR, Elkind MSV, Stone GW, Schwartz A, Ho DD, Bilezikian JP, Landry DW. 2020. Extrapulmonary manifestations of COVID-19. Nat Med 26:1017–1032. doi:10.1038/s41591-020-0968-3. 32651579 PMC11972613

[2] Zheng KI, Feng G, Liu WY, Targher G, Byrne CD, Zheng MH. 2021. Extrapulmonary complications of COVID-19: a multisystem disease? J Med Virol 93:323–335. doi:10.1002/jmv.26294. 32648973 PMC7405144

[3] Corman VM, Landt O, Kaiser M, Molenkamp R, Meijer A, Chu DK, Bleicker T, Brünink S, Schneider J, Schmidt ML, Mulder DG, Haagmans BL, van der Veer B, van den Brink S, Wijsman L, Goderski G, Romette J-L, Ellis J, Zambon M, Peiris M, Goossens H, Reusken C, Koopmans MP, Drosten C. 2020. Detection of 2019 novel coronavirus (2019-nCoV) by real-time RT-PCR. Euro Surveill 25:2000045. doi:10.2807/1560-7917.ES.2020.25.3.2000045. PMC6988269 31992387

[4] van Kampen JJA, van de Vijver DAMC, Fraaij PLA, Haagmans BL, Lamers MM, Okba N, van den Akker JPC, Endeman H, Gommers DAMPJ, Cornelissen JJ, Hoek RAS, van der Eerden MM, Hesselink DA, Metselaar HJ, Verbon A, de Steenwinkel JEM, Aron GI, van Gorp ECM, van Boheemen S, Voermans JC, Boucher CAB, Molenkamp R, Koopmans MPG, Geurtsvankessel C, van der Eijk AA. 2020. Shedding of infectious virus in hospitalized patients with coronavirus disease-2019 (COVID-19): duration and key determinants. medRxiv https://www.medrxiv.org/content/10.1101/2020.06.08.20125310v1. 10.1038/s41467-020-20568-4 PMC7801729 33431879

[5] Hogan CA, Stevens BA, Sahoo MK, Huang C, Garamani N, Gombar S, Yamamoto F, Murugesan K, Kurzer J, Zehnder J, Pinsky BA. 2021. High frequency of SARS-CoV-2 RNAemia and association with severe disease. Clin Infect Dis 72:e291–e295. doi:10.1093/cid/ciaa1054. 32965474 PMC7543277

[6] Fajnzylber J, Regan J, Coxen K, Corry H, Wong C, Rosenthal A, Worrall D, Giguel F, Piechocka-Trocha A, Atyeo C, Fischinger S, Chan A, Flaherty KT, Hall K, Dougan M, Ryan ET, Gillespie E, Chishti R, Li Y, Jilg N, Hanidziar D, Baron RM, Baden L, Tsibris AM, Armstrong KA, Kuritzkes DR, Alter G, Walker BD, Yu X, Li JZ, The Massachusetts Consortium for Pathogen Readiness. 2020. SARS-CoV-2 viral load is associated with increased disease severity and mortality. Nat Commun 11:5493. doi:10.1038/s41467-020-19057-5. 33127906 PMC7603483

[7] Bermejo-Martin JF, González-Rivera M, Almansa R, Micheloud D, Tedim AP, Domínguez-Gil M, Resino S, Martín-Fernández M, Ryan Murua P, Pérez-García F, Tamayo L, Lopez-Izquierdo R, Bustamante E, Aldecoa C, Gómez JM, Rico-Feijoo J, Orduña A, Méndez R, Fernández Natal I, Megías G, González-Estecha M, Carriedo D, Doncel C, Jorge N, Ortega A, de la Fuente A, del Campo F, Fernández-Ratero JA, Trapiello W, González-Jiménez P, Ruiz G, Kelvin AA, Ostadgavahi AT, Oneizat R, Ruiz LM, Miguéns I, Gargallo E, Muñoz I, Pelegrin S, Martín S, García Olivares P, Cedeño JA, Ruiz Albi T, Puertas C, Berezo JÁ, Renedo G, Herrán R, Bustamante-Munguira J, Enríquez P, Cicuendez R, et al. 2020. Viral RNA load in plasma is associated with critical illness and a dysregulated host response in COVID-19. Crit Care 24:691. doi:10.1186/s13054-020-03398-0. 33317616 PMC7734467

[8] Chen X, Zhao B, Qu Y, Chen Y, Xiong J, Feng Y, Men D, Huang Q, Liu Y, Yang B, Ding J, Li F. 2020. Detectable serum severe acute respiratory syndrome coronavirus 2 viral load (RNAemia) is closely correlated with drastically elevated interleukin 6 level in critically ill patients with coronavirus disease 2019. Clin Infect Dis 71:1937–1942. doi:10.1093/cid/ciaa449. 32301997 PMC7184354

[9] Wichmann D, Sperhake J-P, Lütgehetmann M, Steurer S, Edler C, Heinemann A, Heinrich F, Mushumba H, Kniep I, Schröder AS, Burdelski C, de Heer G, Nierhaus A, Frings D, Pfefferle S, Becker H, Bredereke-Wiedling H, de Weerth A, Paschen H-R, Sheikhzadeh-Eggers S, Stang A, Schmiedel S, Bokemeyer C, Addo MM, Aepfelbacher M, Püschel K, Kluge S. 2020. Autopsy findings and venous thromboembolism in patients with COVID-19: a prospective cohort study. Ann Intern Med 173:268–277. doi:10.7326/M20-2003. 32374815 PMC7240772

[10] Braun F, Lütgehetmann M, Pfefferle S, Wong MN, Carsten A, Lindenmeyer MT, Nörz D, Heinrich F, Meißner K, Wichmann D, Kluge S, Gross O, Pueschel K, Schröder AS, Edler C, Aepfelbacher M, Puelles VG, Huber TB. 2020. SARS-CoV-2 renal tropism associates with acute kidney injury. Lancet 396:597–598. doi:10.1016/S0140-6736(20)31759-1. 32818439 PMC7431179

[11] Borczuk AC, Salvatore SP, Seshan SV, Patel SS, Bussel JB, Mostyka M, Elsoukkary S, He B, Del Vecchio C, Fortarezza F, Pezzuto F, Navalesi P, Crisanti A, Fowkes ME, Bryce CH, Calabrese F, Beasley MB. 2020. COVID-19 pulmonary pathology: a multi-institutional autopsy cohort from Italy and New York City. Mod Pathol 33:2156–2168. doi:10.1038/s41379-020-00661-1. 32879413 PMC7463226

[12] Schurink B, Roos E, Radonic T, Barbe E, Bouman CSC, de Boer HH, de Bree GJ, Bulle EB, Aronica EM, Florquin S, Fronczek J, Heunks LMA, de Jong MD, Guo L, Du Long R, Lutter R, Molenaar PCG, Neefjes-Borst EA, Niessen HWM, van Noesel CJM, Roelofs JJTH, Snijder EJ, Soer EC, Verheij J, Vlaar APJ, Vos W, van der Wel NN, van der Wal AC, van der Valk P, Bugiani M. 2020. Viral presence and immunopathology in patients with lethal COVID-19: a prospective autopsy cohort study. Lancet Microbe 1:e290–e299. doi:10.1016/S2666-5247(20)30144-0. 33015653 PMC7518879

[13] Mangalmurti N, Hunter CA. 2020. Cytokine storms: understanding COVID-19. Immunity 53:19–25. doi:10.1016/j.immuni.2020.06.017. 32610079 PMC7321048

[14] Del Valle DM, Kim-Schulze S, Huang H-H, Beckmann ND, Nirenberg S, Wang B, Lavin Y, Swartz TH, Madduri D, Stock A, Marron TU, Xie H, Patel M, Tuballes K, Van Oekelen O, Rahman A, Kovatch P, Aberg JA, Schadt E, Jagannath S, Mazumdar M, Charney AW, Firpo-Betancourt A, Mendu DR, Jhang J, Reich D, Sigel K, Cordon-Cardo C, Feldmann M, Parekh S, Merad M, Gnjatic S. 2020. An inflammatory cytokine signature predicts COVID-19 severity and survival. Nat Med 26:1636–1643. doi:10.1038/s41591-020-1051-9. 32839624 PMC7869028

[15] Zhang X, Tan Y, Ling Y, Lu G, Liu F, Yi Z, Jia X, Wu M, Shi B, Xu S, Chen J, Wang W, Chen B, Jiang L, Yu S, Lu J, Wang J, Xu M, Yuan Z, Zhang Q, Zhang X, Zhao G, Wang S, Chen S, Lu H. 2020. Viral and host factors related to the clinical outcome of COVID-19. Nature 583:437–440. doi:10.1038/s41586-020-2355-0. 32434211

[16] Jøntvedt Jørgensen M, Holter JC, Christensen EE, Schjalm C, Tonby K, Pischke SE, Jenum S, Skeie LG, Nur S, Lind A, Opsand H, Enersen TB, Grøndahl R, Hermann A, Dudman S, Muller F, Ueland T, Mollnes TE, Aukrust P, Heggelund L, Holten AR, Dyrhol-Riise AM. 2020. Increased interleukin-6 and macrophage chemoattractant protein-1 are associated with respiratory failure in COVID-19. Sci Rep 10:21697. doi:10.1038/s41598-020-78710-7. 33303843 PMC7729930

[17] Leisman DE, Ronner L, Pinotti R, Taylor MD, Sinha P, Calfee CS, Hirayama AV, Mastroiani F, Turtle CJ, Harhay MO, Legrand M, Deutschman CS. 2020. Cytokine elevation in severe and critical COVID-19: a rapid systematic review, meta-analysis, and comparison with other inflammatory syndromes. Lancet Respir Med 8:1233–1244. doi:10.1016/S2213-2600(20)30404-5. 33075298 PMC7567529

[18] Gupta S, Madhyastha R, Hamed F, Balkis M, El Nakeidy W, Attallah N. 2020. Tocilizumab use in a chronic hemodialysis patient for the management of COVID-19-associated pneumonia and acute respiratory distress syndrome. Case Rep Nephrol 2020:8829309. doi:10.1155/2020/8829309. 33299621 PMC7682475

[19] Stone JH, Frigault MJ, Serling-Boyd NJ, Fernandes AD, Harvey L, Foulkes AS, Horick NK, Healy BC, Shah R, Bensaci AM, Woolley AE, Nikiforow S, Lin N, Sagar M, Schrager H, Huckins DS, Axelrod M, Pincus MD, Fleisher J, Sacks CA, Dougan M, North CM, Halvorsen Y-D, Thurber TK, Dagher Z, Scherer A, Wallwork RS, Kim AY, Schoenfeld S, Sen P, Neilan TG, Perugino CA, Unizony SH, Collier DS, Matza MA, Yinh JM, Bowman KA, Meyerowitz E, Zafar A, Drobni ZD, Bolster MB, Kohler M, D’Silva KM, Dau J, Lockwood MM, Cubbison C, Weber BN, Mansour MK. 2020. Efficacy of tocilizumab in patients hospitalized with Covid-19. N Engl J Med 383:2333–2344. doi:10.1056/NEJMoa2028836. 33085857 PMC7646626

[20] CORIMUNO-19 Collaborative group. 2021. Effect of anakinra versus usual care in adults in hospital with COVID-19 and mild-to-moderate pneumonia (CORIMUNO-ANA-1): a randomised controlled trial. Lancet Respir Med 9:295–304. doi:10.1016/S2213-2600(20)30556-7. 33493450 PMC7825875

