# Supplementary material for: Temporal Kinetics of RNAemia and Associated Systemic Cytokines in Hospitalized COVID-19 Patients
Source: mSphere. 2021 May 28;6(3):e00311-21. doi: 10.1128/mSphere.00311-21 (PMC8265646; doi:10.1128/mSphere.00311-21)
Supplement: TEXT S1 [file msphere.00311-21-s0001.docx]

**Supplemental Materials and Methods, figures and tables for:**

**Temporal kinetics of RNAemia and associated systemic cytokines in hospitalized COVID-19 patients**

Debby van Riel^1^, Carmen W.E. Embregts^1^, Gregorius J. Sips^1,2^, Johannes P.C. van den Akker^3^, Henrik Endeman ^3^, Els van Nood^2^, Mathijs Raadsen^1^, Lisa Bauer^1^, Jeroen van Kampen^1^, Richard Molenkamp^1^, Marion Koopmans^1^, David van de Vijver^1^ and Corine H. GeurtsvanKessel^1^.

^1^Department of Viroscience; ^2^Medical Microbiology and Infectious Diseases ^3^ Department of Intensive Care; Erasmus MC, Rotterdam, The Netherlands

**Materials and methods:**

**Patient specimen and data collection**

Diagnostic respiratory and serum samples of COVID-19 patients admitted to Erasmus are sent to the unit of clinical virology, Viroscience department, Erasmus MC. In 20 patients admitted in March and April 2020, we performed qPCRs on serum samples collected for diagnostic purposes during admission, as previously described (1). Other available diagnostic results (serology, virus culture and qPCR on respiratory tract specimen) were extracted from our diagnostic laboratory information management system. In addition, following information was extracted from the electronic patient files: date of onset of symptoms, disease severity (hospitalized on ICU with mechanical ventilation, hospitalized on ICU with oxygen therapy, hospitalized to ward with oxygen therapy, hospitalized to ward without oxygen therapy), whether the patients were still alive or not when they were discharged.

**Medical ethical approval**

All patient specimen and data used in this study were collected in the context of routine clinical patient care. Additional analyses were performed only on surplus of patient material collected in the context of routine clinical patient care. Our institutional review board approved the use of these data and samples (METC-2015-306). METC-2015-306 is a generic protocol to study viral diseases. Informed consent was waived by the privacy knowledge office of the Erasmus MC.

**Cytometric bead assay**

Systemic cytokines were quantified in all patients’ sera up to 21 dpd. Cytokines were also quantified in sera of healthy donors (n=18) as a control group. Sera were analysed in duplo using the 13-plex Human essential immune Legendplex panel (Biolegend). A standard cytokine cocktail was taken along in duplo to allow for quantification of the cytokine concentrations. The cytometric bead assay was performed on a FACSLyric (BD) and the data were analysed using the Legendplex data analysis software. Detection limits were based on the standard cytokine cocktail and were determined by the Legendplex data analysis software. Limits of detection for the individual cytokines are as follows (in pg/mL): IFN-γ (3), IL-1 β (0.82), IL-2 (2.13), IL-6 (10), IL-8 (3.36), IL-10 (1.66), IL-17A (3.24), IP-10 (49.91), MCP-1 (5.97), TNF-α (1.15), IL-4 (16.27), IL12p70 (2.86), TGF-β (25.65).

**Analyses**

Statistical analysis was performed using R version 4.0.3 and the geepack package (2). Associations between presence of RNAemia and a Ct <30 in respiratory samples and a dpd <11 were determined after dichotomizing the values and evaluating the fit according to the quasi-likelihood under the independence criterion (QIC) by generalized estimating equations corrected for multiple samples per patient. Associations between individual cytokines and RNAemia, a ct <30 in respiratory samples, critical disease and a fatal outcome were assessed using generalized estimating equations. All analyses were corrected for multiple samples per patient by clustering the data based on patient ID. Associations of cytokines with a p-value of <0.1 were subjected to a multivariate analysis. In the specific case of comparing patient samples to samples of healthy controls, only univariate analyses were performed. The low expression of various cytokines in the healthy control group resulted in a strong correlation of factors (cytokines), leading to the violation of the assumption of the absence of multicollinearity. Due to this violation, multivariate analyses could not be performed.

**References**

1. van Kampen J.J.A vdVDAMC, Fraaij P.L.A., Haagmans B.L., Lamers M.M., Okba N, van den Akker J.P.C., Endeman H., Gommers D.A.M.P.J., Cornelissen J.J., Hoek R.A.S., van der Eerden M.M, Hesselink D.A., Metselaar H.J., Verbon A., de Steenwinkel J.E.M, Aron G.I., van Gorp E.C.M., van Boheemen S., Voermans J.C., Boucher C.A.B., Molenkamp R., Koopmans M.P.G., Geurtsvankessel C., van der Eijk A.A. Shedding of infectious virus in hospitalized patients with coronavirus disease-2019 (COVID-19): duration and key determinants. MedRxiv. 2020.

2. Søren Højsgaard UH, Jun Yan. The R Package geepack for Generalized Estimating Equations. Journal of Statistical Software. 2006;15(2).
