# Supplementary material for: Temporal Kinetics of RNAemia and Associated Systemic Cytokines in Hospitalized COVID-19 Patients
Source: mSphere. 2021 May 28;6(3):e00311-21. doi: 10.1128/mSphere.00311-21 (PMC8265646; doi:10.1128/mSphere.00311-21)
Supplement: TABLE S1 [file msphere.00311-21-st001.docx]

***Table S1****: Information of patients and healthy donors included in the study*

|  | | Moderate/Severe  disease | Critical  disease | Total | Healthy donors |
| --- | --- | --- | --- | --- | --- |
| Total | | 10 | 10 | 20 | 18 |
| Gender | **Male Female** | 9  1 | 7  3 | 16  4 | 7  11 |
| Age* | **Mean**  **Range** | 60.8  25-84 | 59.3  17-84 | 60.1  17-84 | 34  24-63 |
| BMI* | **Mean**  **Range** | 26.3  19-33 | 29.2  24-38 | 28  19-38 | NA |
| Outcome | **Death** | 0 | 5 | 5 |  |

* no significant differences between patients with moderate/severe and critical disease.
