# Supplementary material for: Temporal Kinetics of RNAemia and Associated Systemic Cytokines in Hospitalized COVID-19 Patients
Source: mSphere. 2021 May 28;6(3):e00311-21. doi: 10.1128/mSphere.00311-21 (PMC8265646; doi:10.1128/mSphere.00311-21)
Supplement: TABLE S2 [file msphere.00311-21-st002.docx]

***Table S2:*** *Detection of viral RNA serum of patients with moderate/severe or critical disease. Analyses are done on the total number of samples, and on samples from 1-10 days post disease onset (dpd) and >10 dpd.*

|  | | Patient with moderate or severe  disease  (samples) | Patient with critical disease  (samples) | Total number  of patients  (samples) |
| --- | --- | --- | --- | --- |
| Number of samples | Total  1-10 dpd  >10 dpd | 10 (57)  6 (17)  10 (40) | 10 (119)  8 (35)  9 (84) | 20 (176)  14 (52)  19 (124) |
| PCR positive samples  (E-gene) | Total  1-10 dpd  >10 dpd* | 5 (10)  4 (8)  2 (2) | 9 (51)  7 (23)  7 (28) | 14 (61)  11 (31)  9 (30) |
| % positive  samples | Total  1-10 dpd  >10 dpd | 50 (18)  67 (47)  20 (5) | 90 (43)  88 (66)  78 (33) | 70 (35)  79 (60)  47 (24) |
| RNAemia | Mean spd**  Range dpd | 7  2-12 | 11.6  4-21 | 10.7  2-21 |
| Leukocytes  (10^9/mL) | Median *  Range | 6.9  2.6-18.7 | 9.5  2.8-39 | 8.2  2.6-39 |
|  | |  |  |  |

* p<0.05 between patients with moderate/severe and critical disease

** p<0.01 between patients with moderate/severe and critical disease
