# Supplementary figures and images for: Temporal Kinetics of RNAemia and Associated Systemic Cytokines in Hospitalized COVID-19 Patients
Source: mSphere. 2021 May 28;6(3):e00311-21. doi: 10.1128/mSphere.00311-21 (PMC8265646; doi:10.1128/mSphere.00311-21)

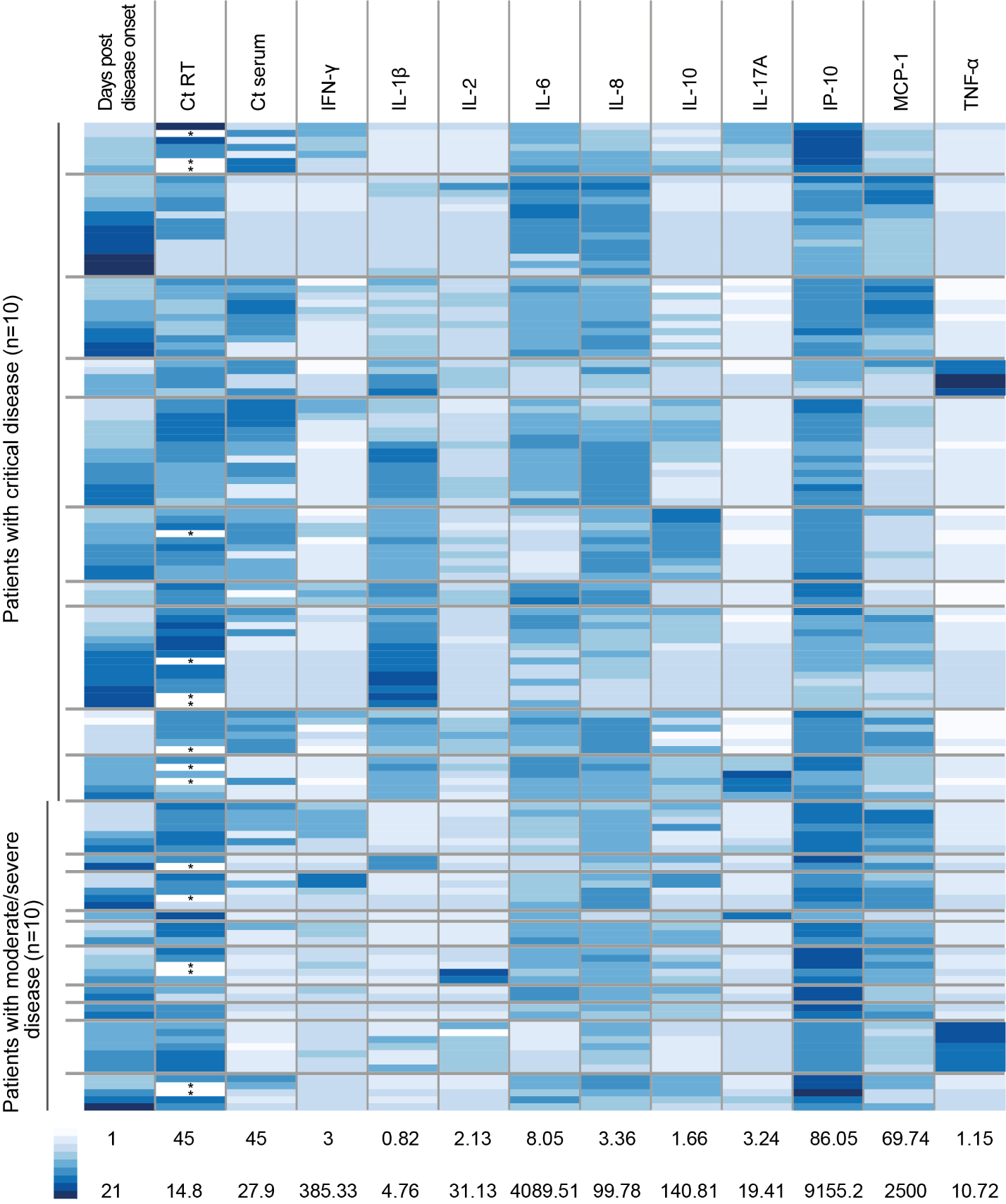

Supplement: FIG S1 [file msphere.00311-21-sf001.tif]

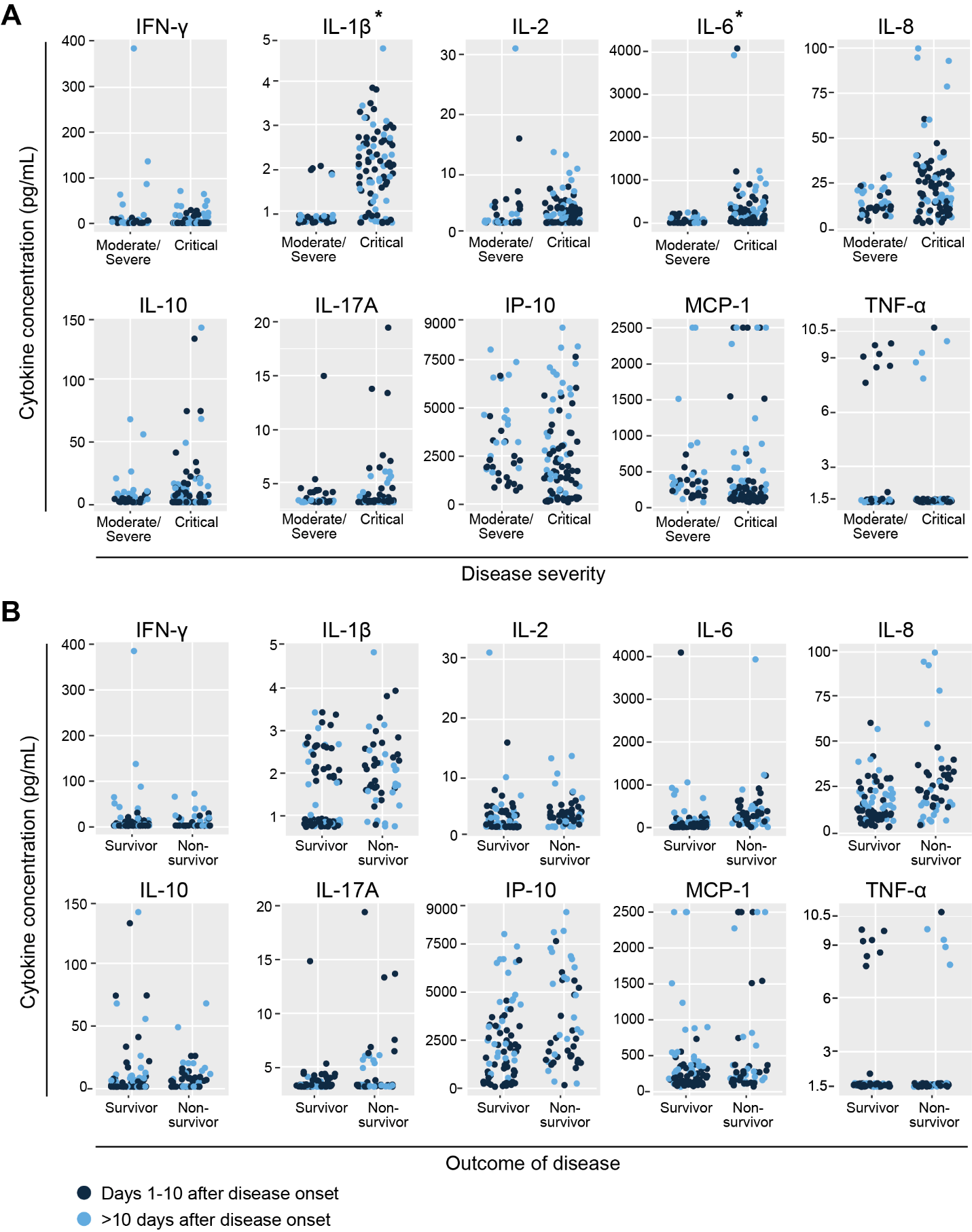

Supplement: FIG S2 [file msphere.00311-21-sf002.tif]

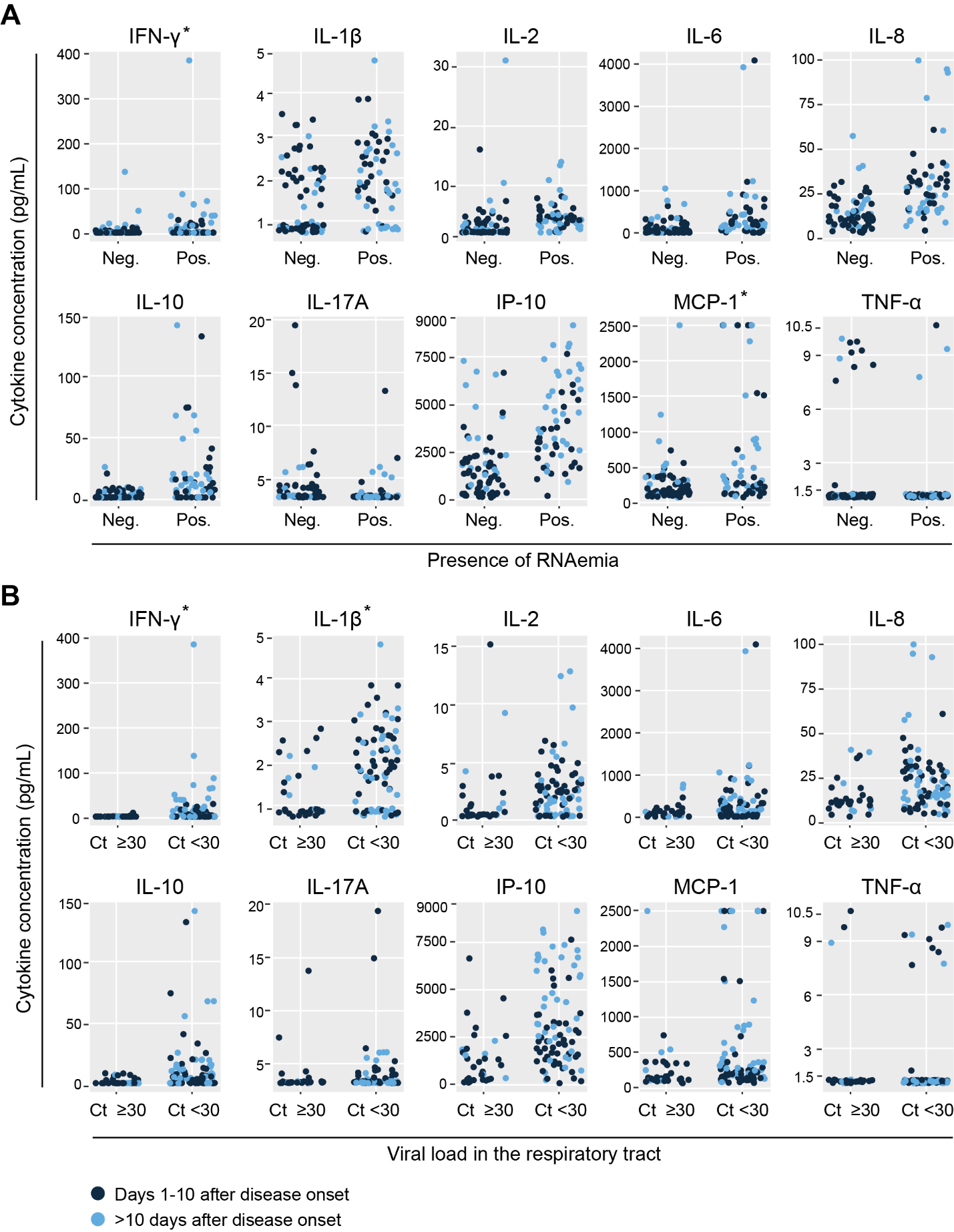

Supplement: FIG S3 [file msphere.00311-21-sf003.tif]
