# Supplementary material for: Temporal Kinetics of RNAemia and Associated Systemic Cytokines in Hospitalized COVID-19 Patients
Source: mSphere. 2021 May 28;6(3):e00311-21. doi: 10.1128/mSphere.00311-21 (PMC8265646; doi:10.1128/mSphere.00311-21)
Supplement: TABLE S3 [file msphere.00311-21-st003.docx]

***Table S3:*** *Concentrations of individual cytokines in serum of healthy donors, and univariate analysis of cytokine concentrations in serum of healthy donors compared to SARS-CoV-2 patients, moderate/severe patients, critical patients and RNAemia positive serum samples. Univariate generalized estimated equations were performed on the individual cytokines (log10 transformed) and differences with a p <0.05 were regarded as significant.*

|  | **Healthy donors** | **SARS-CoV-2 patients** | | | |
| --- | --- | --- | --- | --- | --- |
|  | **Cytokine concentration** | **All patients** | **Moderate/severe** | **Critical** | **RNAemia+** |
|  | **Median pg/mL (range)** | **p-value of univariate analysis** | | | |
| **IFN-γ** | 4.3 (3-117.3) | 0.778 | 0.270 | 0.786 | 0.786 |
| **IL-1β** | 2.8 (0.9-45) | **0.001** | **< 0.001** | **0.011** | **0.011** |
| **IL-2** | 2.1 (2.1-21.6) | 0.450 | 0.860 | 0.300 | 0.300 |
| **IL-6** | 10 10-61.4) | **0.006** | 0.099 | **< 0.001** | **< 0.001** |
| **IL-8** | 8.3 (4-54.6) | **0.008** | **0.045** | **0.004** | **0.004** |
| **IL-10** | 2.7 (1.7-47.3) | 0.158 | 0.260 | 0.093 | 0.093 |
| **IL-17A** | 4.9 (3.2-47.4) | **0.038** | 0.083 | **0.026** | **0.026** |
| **IP-10** | 490.2 (50-1640.6) | **0.003** | **< 0.001** | **0.001** | **0.001** |
| **MCP-1** | 135.2 (6-217.9) | **< 0.001** | **< 0.001** | **< 0.001** | **< 0.001** |
| **TNF-α** | 14.8 (1.3-453.2) | 0.060 | **0.031** | **< 0.001** | **< 0.001** |
